# After Treatment in Necrosis

**Published:** 1868-01-01

**Authors:** 


### AFTER-TREATMENT IN NECROSIS.

BY PROFESSOR GUNN.

At an early period in my professional experience, I encountered the necessity of secondary operations in certain cases of Necrosis. After the usual operation and dressing, while the limb would improve in general appearance, the wound would fail to completely heal, or, if it did heal, it would reopen in a short time. Examination with the probe would detect a piece of necrosed bone, which usually was insignificant in size, but which prevented healing, and necessitated a new opening of the wound for its removal.

Having encountered this experience in several instances, I was led to an examination of some specimens of this disease when the sequestra were contained in separate compartments of the capsula sequestralis, but between which there was a free communication.

In an operation on such a case, the surgeon would probably open the main chamber or compartment, remove the main sequestrum, and fail to detect one or more, which were smaller, and lodged in small side alcoves or compartments, and which, after the removal of the main piece, would naturally work, in the course of a few days, into the place which it had occupied. It is a well recognized fact, that reparative nutrition tends to the pushing out or off of foreign material. Thus, these remaining sequestra would come to occupy the place of the main sequestrum on their route out of the body.

To secure their final expulsion without an additional operation, I devised the plan of introducing into the opening through the walls of the capsula sequestralis, made for the removal of the main sequestrum, a plug or tent of white wax, which fitted the opening pretty accurately, without so completely filling it as to obstruct the flow of pus. This plug is removed daily, it and the wound are cleansed, when it is reinserted. At each dressing, the wound is explored with the finger, and when additional sequestra are detected, they are removed. After they have all come away, or, if they have all been removed at the time of the operation, the fact will be indicated by the gradual filling of the chamber by firm, healthy granulations, after which time the plug may have a piece cut off from its bottom at each dressing, until it is all cut away, and the wound has healed to the surface.

Under this plan of treatment, it has often been my experience to detect and remove one or more pieces of dead bone within the first two weeks after the operation, which had eluded discovery at that time. The tardy growth of the granulations compelled by this style of dressing, secures a firm and healthy healing progress from the very bottom of the wound.

